# Optimizing pentose utilization in yeast: the need for novel tools and approaches

**DOI:** 10.1186/1754-6834-3-24

**Published:** 2010-11-16

**Authors:** Eric Young, Sun-Mi Lee, Hal Alper

**Affiliations:** 1 Department of Chemical Engineering, The University of Texas at Austin, 1 University Station, C0400, Austin, Texas 78712, USA; 2 Water Environment Center, Korea Institute of Science and Technology, 39-1 Hawolgok-dong, Seongbuk-gu, Seoul 136-791, Korea

## Abstract

Hexose and pentose cofermentation is regarded as one of the chief obstacles impeding economical conversion of lignocellulosic biomass to biofuels. Over time, successful application of traditional metabolic engineering strategy has produced yeast strains capable of utilizing the pentose sugars (especially xylose and arabinose) as sole carbon sources, yet major difficulties still remain for engineering simultaneous, exogenous sugar metabolism. Beyond catabolic pathways, the focus must shift towards non-traditional aspects of cellular engineering such as host molecular transport capability, catabolite sensing and stress response mechanisms. This review highlights the need for an approach termed 'panmetabolic engineering', a new paradigm for integrating new carbon sources into host metabolic pathways. This approach will concurrently optimize the interdependent processes of transport and metabolism using novel combinatorial techniques and global cellular engineering. As a result, panmetabolic engineering is a whole pathway approach emphasizing better pathways, reduced glucose-induced repression and increased product tolerance. In this paper, recent publications are reviewed in light of this approach and their potential to expand metabolic engineering tools. Collectively, traditional approaches and panmetabolic engineering enable the reprogramming of extant biological complexity and incorporation of exogenous carbon catabolism.

## Introduction

Conversion of lignocellulosic biomass into fuels and chemicals is an attractive, sustainable alternative to traditional petroleum refining [1]. Accomplishing this goal using fungal or microbial hosts requires optimized cellular systems [2]. Since its inception, metabolic engineering has served as an effective platform approach to optimize and control small molecule production in cells [3-8]. Moreover, recent developments in genetic approaches have advanced the metabolic engineering toolbox beyond overexpression and knockout techniques [9-13]. This progress is evinced by the wide variety of secondary metabolite and novel products engineered for production in cells [14-21]. It is anticipated that metabolic engineering approaches can help solve the challenges of biofuel production [22-24]. However, these approaches have all traditionally focused on rerouting cellular metabolism using standard native sugars as carbon sources.

Lignocellulosic biomass presents new challenges for the metabolic engineering paradigm [25,26]. Although biomass is chiefly composed of glucose, the pentose sugars D-xylose and L-arabinose can also constitute significant fractions (up to 20%) that must be converted [27]. Even though many organisms are capable of natively converting these sugars, the most commonly selected organism is baker's yeast (*Saccharomyces cerevisiae*). The genetic tractability, widespread industrial use and endogenous ethanol production capacity of yeast motivate its use [28]; however, baker's yeast must be engineered to convert xylose and arabinose. Traditional pathway engineering approaches have enabled xylose and arabinose catabolism in yeast, but continued optimization of these strains requires novel metabolic engineering tools and strategies. Specifically, novel approaches should target and exploit additional cellular mechanisms influencing metabolic pathways, such as molecular transport, catabolite sensing and cellular tolerances.

In this review, we propose simultaneous transport and metabolic engineering, including new global cellular metabolic engineering techniques, as a powerful approach for the development of an economical hexose and pentose cofermenting yeast. As this review will illustrate, molecular transport and metabolic pathway engineering have conventionally been studied in isolation despite their intricate interdependence. To efficiently arrive at an optimized strain, metabolic engineering tools must be expanded to modify multiple interdependent steps, an approach we term 'panmetabolic engineering'. Metabolic engineering tools must also expand to incorporate recent breakthroughs in modifying catabolite sensing and in increasing cellular tolerances to significantly affect biofuel-producing organisms. Once developed, these tools will enable the addition of other substrates to the yeast carbon source portfolio. Therefore, the scope of this review is to discuss recent literature concerning pentose transport and metabolism in yeast, and to suggest a path forward for engineering nonnative carbon source metabolism in organisms.

### Engineering exogenous pentose sugar utilization in yeast

#### Pathway assembly by heterologous gene expression

Baker's yeast is an ideal industrial fermentation host; however, it possesses little to no xylose or arabinose metabolic capability. Interestingly, the *S. cerevisiae *genome is encoded with a putative xylose metabolic pathway, although the expression level of these genes are often too low to permit growth on xylose as the sole carbon source [29-31]. Recent work suggests that some strains of *Saccharomyces *may possess a latent oxidoreductase pathway with an active xylitol dehydrogenase [32]. However, this pathway was shown to be repressed by other putative xylitol dehydrogenases, illustrating unique and potentially *trans*-acting control mechanisms. Even so, yeast lack effective xylose and arabinose utilization pathways, and therefore require heterologous complementation or significant genetic modification.

**Figure 1:**
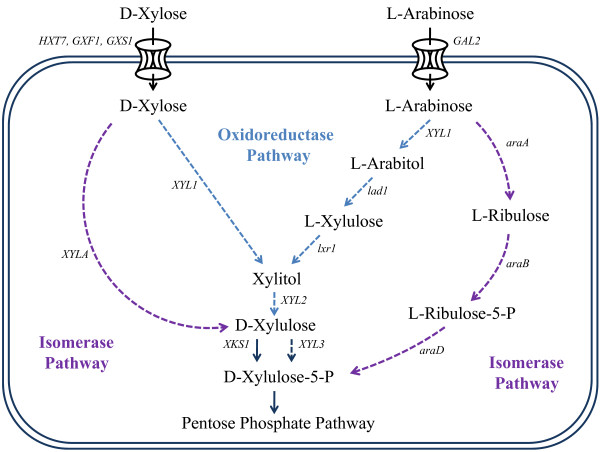
**Successful exogenous transport and metabolic pathways introduced in *S. cerevisiae***. Reported improvements of pentose utilization in yeast (as described in the text) are depicted in this schematic. **(A) **Bacterial xylose isomerase pathway; **(B) **fungal xylose oxidoreductase pathway; **(C) **Fungal arabinose oxidoreductase pathway; **(D) **Bacterial arabinose isomerase pathway. The genes used to accomplish the enzymatic step are italicized. Heterologous steps are indicated by dashed lines.

The advent of recombinant DNA technology enabled the transfer of genes from native pentose catabolizing organisms into baker's yeast, thus facilitating novel pentose catabolism [33-36]. Two types of pentose pathways have been constructed in yeast: the oxidoreductase pathway and the isomerase pathway (Figure 1). Both xylose and arabinose can be metabolized through each of these pathways, although arabinose assimilation involves additional steps in both cases [37]. All four possible pathway variants have been previously constructed [37-40], and all feed into native yeast metabolism via D-xylulose or D-xylulose-5-phosphate (P). Once converted to xylulose 5-P, these sugars are further metabolized through the native pentose phosphate pathway (PPP). The following sections give a brief review of these pathways. Combined with molecular transporter engineering, they form the foundation of the panmetabolic engineering described in more detail later.

#### Oxidoreductase pathways

The pentose oxidoreductase pathways are conserved between certain species of native fungi, and employ common enzymes and redox cofactors to catalyze substrate conversion. The xylose oxidoreductase pathway was the first heterologous pentose pathway constructed in baker's yeast [38]. In this pathway, depicted in Figure 1, xylose is reduced to xylitol by an aldose reductase (AR), then xylitol is oxidized to xylulose by xylitol dehydrogenase (XDH). The AR most commonly used is encoded by *Pichia stipitis XYL1 *(xylose reductase, XR) which prefers the cofactor NADPH over NADH. The XDH is encoded by *P. stipitis XYL2*, which is NAD^+ ^dependent [41].

The L-arabinose oxidoreductase pathway was also the first variant constructed in baker's yeast for arabinose conversion [42]. Figure 1 illustrates that the two enzymes from the xylose oxidoreductase pathway AR and XDH serve as catalysts of the first and last reactions, respectively. The remaining two steps have recently been constructed using two genes from the fungus *Trichoderma reesei *(*Hypocrea jecorina*). The first gene, *lad1*, encodes an arabitol 4-dehydrogenase (ADH) which reduces arabitol to L-xylulose using NADPH as a cofactor [43]. The second is an L-xylulose reductase (XR) encoded by *lxr1*, which reduces L-xylulose to D-xylulose using NAD^+ ^as a cofactor [42].

The D-xylulose from both the xylose and arabinose variants is then utilized by native metabolic steps, culminating in the production of biomass, carbon dioxide and ethanol.

It is important to recognize the cofactor imbalances of the arabinose and xylose oxidoreductase pathways. This imbalance is thought to limit theoretical and actual pentose conversion by yeast [25,44]. Whereas the enzymes discussed above alone are sufficient to enable xylose or arabinose metabolism in yeast, the xylulokinase gene from *P. stipitis *(*XYL3*) is often complemented to create a complete heterologous pathway and further reduce xylitol production [28,45,46].

#### Isomerase pathways

In contrast to the pentose oxidoreductase pathway, the isomerase pathway variants require no cofactors. The pathway is native to bacterial species and to rare yeasts. The heterologous xylose isomerase pathway minimally consists of one enzyme, xylose isomerase (XI), which directly converts xylose to xylulose (Figure 1). Because most XIs are native to bacteria, difficulties for heterologous expression in yeast exist [47], yet recent work has demonstrated functional bacterial XI pathways [39,48]. In addition, bioprospecting has yielded functional heterologous XIs isolated from rare fungi [49,50]. As with the oxidoreductase pathway, the complementation of a xylulokinase can further improve yields and assimilation rates. This pathway has improved ethanol conversion yields over the oxidoreductase pathway; however, strains have lower growth rates and sugar uptake rates, as discussed later. Nevertheless, this pathway is attractive because of its lack of cofactor imbalance.

Whereas the xylose isomerase pathway involves one step, the arabinose isomerase pathway consists of three. First, an arabinose isomerase encoded by *araA *converts arabinose to ribulose. Second, a ribulokinase encoded by *araB *phosphorylates ribulose to ribulose 5-P. Third, ribulose-5-P-4-epimerase, encoded by *araD*, catalyzes the final epimerization of ribulose 5-P to D-xylulose-5-P, an intermediate in the PPP. Two variations of this pathway have been constructed in yeast using distinct sets of heterologous genes. The first set uses *Bacillus subtilis araA *along with *Escherichia coli araB *and *araD *[40]. The second relies on the expression of *Lactobacillus plantarum araA, araB *and *araD *[51]. However, unlike the other pathways described above, evolutionary engineering was needed in both cases to isolate a yeast strain with an active arabinose isomerase pathway. Thus, only after mutations is a functional arabinose isomerase pathway in yeast possible.

#### Optimization of limiting steps

The work described above establishes viable pentose utilization pathways in baker's yeast, enabling xylose and arabinose to be used as a sole carbon source. However, reported growth rates remain suboptimal for economical production of biofuels from lignocellulosic biomass [2]. This limitation has driven further improvements of the heterologous pathways by both traditional metabolic engineering approaches and heterologous molecular transporter expression.

The following sections review individual efforts to improve internal pathways and transport in yeast. Through this work, limiting steps have been identified and partially alleviated. However, pathway optimization and transporter engineering in isolation has not yet produced an optimal organism. An approach integrating both pathway and transport engineering may produce more industrially viable results.

#### Internal pathway

When the xylose oxidoreductase pathway is expressed in *S. cerevisiae*, several factors limit ethanol production, with the primary one being cofactor imbalance. XR has a higher specificity for NADPH than for NADH (K_m _= 3.2 μmol/l for NADPH and K_m _= 40 μmol/l for NADH) and XDH uses only NAD^+^. In yeast, there is a disparity between the amount and accessibility of intracellular NADPH and NADH for the xylose pathway enzymes [52,53]. This disparity could be due to competition for NAD+ by other endogenous metabolic enzymes or to inefficient xylose pathway enzymes. As a result, high amounts of xylitol may be produced [41]. A severe cofactor imbalance (with two NADPH and two NAD^+ ^required) limits the arabinose oxidoreductase pathway [54]. As a result, this pathway exhibits poor cell growth or little ethanol production, even though the enzymes are actively expressed [36,54].

Modifying the affinity or expression level of XR and XDH in the xylose oxidoreductase pathway has been shown to decrease xylitol production and increase ethanol yield [37,55-57]. However, even with adjusting cofactor preferences, a significant increase in ethanol production has not been achieved [25,56]. Xylitol formation may not be solely a consequence of inappropriate redox balance; some additional metabolic factors in the host may cause its production. Supporting this hypothesis, native *P. stipitis *does not produce significant amounts of xylitol, whereas recombinant *S. cerevisiae *expressing the same genes produces abundant xylitol [53]. Xylitol is still seen in strains expressing a complete *XYL1/2/3 *pathway from *P. stipitis*. Therefore, balancing mechanisms eliminating excess xylitol in *P. stipitis *may not be present in baker's yeast.

In the xylose isomerase pathway, xylose is converted to xylulose through a one step enzyme reaction catalyzed by xylose isomerase [41]. In this redox-neutral process, cofactor imbalance is avoided, no xylitol is produced, and the ethanol yield is much higher than in the oxidoreductase pathway [25,49,50]. However, strains expressing xylose isomerase have low anaerobic xylose consumption and growth rates due to insufficient activity of the heterologous xylose isomerase [35,46,50,53,58]. Moreover, at equilibrium, the isomerization reaction is more favorable for xylose than xylulose formation (ratio of 80:20) [59,60]. Beyond genetic approaches, the equilibrium of the xylose isomerase reaction can be shifted to favor xylulose formation by supplementing with chemicals such as borate [61].

These facts emphasize the need for either increased bioprospecting for more active xylose isomerases or protein engineering to improve performance in yeast. Optimizing expression is also important, and coregulating a xylose isomerase with downstream enzymes may prove effective. Because the xylose isomerase pathway has only recently been engineered in *S. cerevisiae*, an improved xylose isomerase with satisfactory activity may still be discovered or engineered.

Consistent with the xylose isomerase pathway, the activity of arabinose isomerase is insufficient, and the isomerization of arabinose to ribulose is unfavorable [54,62]. In particular, insufficient cell growth and lower ethanol production rates are seen with cells grown on arabinose compared with glucose or even xylose [54]. Therefore, metabolic and protein engineering solutions for increasing the growth rate and shifting the driving force from aldose to ketose could improve both xylose and arabinose assimilation in recombinant *S. cerevisiae*.

Beyond the recombinant pathway steps, additional work in improving pentose utilization has focused on the native PPP. The enzymes xylulokinase (*XKS1 *gene), transaldolase (*TAL1*) and transketolase (*TKL1*) have been supplemented by heterologous or overexpressed native genes. These heterologous genes have typically been imported from *P. stipitis*;, for example, *PsTAL1 *may be expressed to supplement *ScTAL1*[45]. In the case of transaldolase, the *Pichia *version of the enzyme had more advantageous enzyme kinetics than the *Saccharomyces *version. This observation raises the possibility that additional superior PPP enzyme may exist in native xylose consumers. Bioprospecting provides a means of identifying such enzymes.

Taken together, metabolic engineering has been able to construct and improve the pentose utilization pathways in yeast. However, these improvements have yet to produce a strain that allows widespread lignocellulosic biomass conversion to biofuels. Explanations may include host genotype fitness; for example, the putative xylitol dehydrogenases may actually be detrimental to xylose metabolism [32,45]. These and other potential regulatory mechanisms such as protein acetylation patterns or other unknown regulatory factors may be inhibiting pentose utilization [63]. Therefore, more targets and studies are required to advance the field. One of the unique primary targets being examined is the optimization of pentose transport in yeasts.

#### Transport

Sugar transport across the membrane does not significantly limit the endogenous metabolism of sugars such as glucose, although it may limit exogenous pentose metabolism. Without any genetic modifications, baker's yeast will transport pentoses across the cell membrane through one of many native hexose transport proteins: the *S. cerevisiae *proteins Hxt7p, Hxt5p, Hxt4p, Hxt2p, Hxt1p and Gal2p [40,64-66]. However, these proteins have a much higher affinity for their native hexose substrates, which may create unfavorable competitive inhibition and lead to diauxic growth in a hexose-pentose cofermentation. Therefore, dedicated pentose transport provides an opportunity to improve the simultaneous use of hexoses and pentoses.

It has been demonstrated or inferred in several cases that pentose transport accounts for significant pathway flux control [67-69]. Gardonyi *et al. *reported a flux control coefficient of 0.2 for xylose transport in *S. cerevisiae *TMB 3001 (CEN.PK-2 XR/XDH/XKS) regardless of xylose concentration, and rising to 0.5 in *S. cerevisiae *TMB 3206 (TMB 3001 with overexpressed XR) at xylose concentrations below 0.6 g/l [67]. Recently, an evolutionary engineering experiment (aided by continuous culturing in xylose) was performed using an optimized *S. cerevisiae *strain overexpressing six key xylose metabolic enzymes (including XI, XKS, TAL and TKL) [68]. Accumulated mutations over time resulted in greatly altered xylose transport kinetics, doubling V_max _(15.8 to 32 mmol per dry weight per hour) and reducing K_m _by 25% (132 to 99 mmol/lM). From this result, it may be inferred that xylose transport is a limiting step, especially in metabolically optimized strains with higher downstream flux capacity. An additional evolutionary engineering experiment with recombinant *S. cerevisiae *found an increase in expression of the hexose transporter gene *HXT5 *[69], showing that cells choose to increase transport activity when selected on xylose. In terms of L-arabinose transport, it has been demonstrated that the native hexose transporter gene *GAL2 *is essential for pathway function [40], thus implicating the lack of a specific arabinose transporter protein.

The above findings underpin recent work focusing on heterologous transporter protein identification and expression to improve xylose uptake. Heterologous transport proteins from plant, bacteria and other yeasts have been cloned and expressed in recombinant *S. cerevisiae. *However, only the class of transporters native to yeast, the major facilitator superfamily (MFS) [70], has been investigated. Other classes, such as the ATP binding cassette (ABC) transporters and the bacterial phosphoenolpyruvate (PEP) dependent transporters, are unlikely to be effective, because of expression difficulty and high relative energy requirements [71]. Despite ongoing work and bioprospecting, researchers have been unable to isolate or evolve a transport protein with xylose as its highest affinity substrate. Such a transporter would greatly enhance the prospects of a glucose-xylose cofermentation.

The collective results from heterologous pentose transporter expression studies are summarized in Table 1. To date, no heterologous arabinose transport experiments have been conducted, in part because of the recent reconstruction of an arabinose metabolic pathway in yeast. The most successful improvement of xylose transport to date is the expression of *Candida intermedia *PYCC 4715 transport proteins [72]. Both *C. intermedia GXF1 *and *GXS1 *conferred significant improved growth phenotypes in recombinant *S. cerevisiae *when glucose and xylose were used as sole carbon sources. Subsequent work has evaluated coexpression of the two proteins [73] as well as more in-depth fermentation analysis [74]. Both of these transporters are efficient xylose transporters, yet also have a high affinity for glucose. Therefore, they remain primarily hexose transporters, despite the excellent xylose transport characteristics.

**Table 1:** Transport proteins studied for xylose uptake.

Genus	Species	Gene or DNA Library	UniProt	Plasmid	Study	Strain	Xylose transport phenotype
*Arabidopsis*	*thaliana*	Stp2	Q9LNV3	p4H7	[64]	TMB3201	-

*Arabidopsis*	*thaliana*	Stp3	Q8L7R8	p4H7	[64]	TMB3201	-

*Arabidopsis*	*thaliana*	At5g59250	Q0WWW9	p4H7	[64]	TMB3201	-

*Arabidopsis*	*thaliana*	At5g59250	Q0WWW9	pRH145	[75]	BY4727	+

*Arabidopsis*	*thaliana*	At5g17010	Q6AWX0	pRH145	[75]	BY4727	+

*Arabidopsis*	*thaliana*	Seedling cDNA [104]	-	pFL61	[64]	TMB3201	-

*Candida*	*intermedia*	Gxf1	Q2MDH1	YEplac195	[72]	TMB3201	+

*Candida*	*intermedia*	Gxs1	Q2MEV7	YEplac195	[72]	TMB3201	+

*Chlorella*	*kessleri*	Hup1	P15686	p4H7	[64]	TMB3201	-

*Escherichia*	*coli*	XylE	P0AGF4	p4H7	[64]	TMB3201	-

*Pichia*	*stipitis*	SUT1	O94155	YEp24	[105]	RE700	-

*Pichia*	*stipitis*	SUT2	O94151	YEp24	[105]	RE700	-

*Pichia*	*stipitis*	SUT3	Q9UWF5	YEp24	[105]	RE700	-

*Pichia*	*stipitis*	Genomic library [105]	-	YEp24	[64]	TMB3201	-

*Trichoderma*	*reesei*	Xlt1	Q1EG32	pAJ401	[66]	H2219	+*

*Saccharomyces*	*cerevisiae*	HXT1	P32465	pYX212	[66]	H2219	+

*Saccharomyces*	*cerevisiae*	HXT2	P23585	pYX212	[66]	H2219	+

*Saccharomyces*	*cerevisiae*	HXT4	P32467	pYX212	[66]	H2219	+

*Saccharomyces*	*cerevisiae*	HXT7	P39004	pYX212	[66]	H2219	+

*Saccharomyces*	*cerevisiae*	HXT1	P32465	p4H7	[64]	TMB3201	-

*Saccharomyces*	*cerevisiae*	HXT3	P32466	p4H7	[64]	TMB3201	-

*Saccharomyces*	*cerevisiae*	HXT4	P32467	p4H7	[64]	TMB3201	+

*Saccharomyces*	*cerevisiae*	HXT5	P38695	p4H7	[64]	TMB3201	+

*Saccharomyces*	*cerevisiae*	HXT7	P39004	YEpkHXT7	[64]	TMB3201	+

*Saccharomyces*	*cerevisiae*	HXT8	P40886	p4H7	[64]	TMB3201	-

*Saccharomyces*	*cerevisiae*	HXT9	P40885	p4H7	[64]	TMB3201	-

*Saccharomyces*	*cerevisiae*	HXT10	P43581	p4H7	[64]	TMB3201	-

*Saccharomyces*	*cerevisiae*	HXT11	P54862	p426MET25	[64]	TMB3201	-

*Saccharomyces*	*cerevisiae*	HXT13	P39924	p4H7	[64]	TMB3201	-

*Saccharomyces*	*cerevisiae*	HXT14	P42833	p4H7	[64]	TMB3201	-

*Saccharomyces*	*cerevisiae*	HXT15	P54854	p4H7	[64]	TMB3201	-

*Saccharomyces*	*cerevisiae*	GAL2	P13181	pHL125	[64]	TMB3201	+

Several previous studies have evaluated the capacity of selected transport proteins to uptake xylose in yeast.

The strain genotype (described in Table 2) used and the identified xylose phenotype is listed.

*Indicates that mutations (either in the gene or in the host cell) were required for the phenotype to appear.

Heterologous xylose transport phenotypes were observed in yeast expressing *A. thaliana At5g59250 *and *At5g17010 *[75]. However, this observation was not supported by a second study in which *At5g59250 *expression did not confer growth on xylose [64]. These studies, as Table 2 clarifies, did not use the same host strain. In the first study with *At5g59250*, a standard strain of yeast was used whereas in the second, a strain lacking the HXT family of proteins was used. Interactions between membrane proteins, such as those demonstrated above with *GXF1 *and *GXS1 *[73], may explain this discrepancy. In this regard, the *A. thaliana *proteins may potentially act as sensors or activators of HXT family transporters. These results illustrate the importance of genotype on transporter characterization studies. Moreover, they emphasize the need for simultaneous optimization of both transport processes and metabolic pathways.

**Table 2:** Various host strains used to analyze transporter function.

Strain	Relevant genotype	Other plasmids (promoter and gene)
TMB3201	Δhxt1-17 Δgal2 Δstl1 Δagt1 Δmph2 Δmph3 his3-Δ1::YIpXR/XDH/XK	-

BY4727	MATα his3Δ200 leu2Δ0 lys2Δ0 met15Δ0 trp1Δ63 ura3Δ0	P_ADH1_-PsXYL2, P_PGK1_-PsXYL1, P_HXT7_-ScXKS1-T_HXT7_

H2219	Δhxt1-7 Δgal2 ura3-Δ1::XR/XDH his3-Δ1::XK	-

RE700	Δhxt1-7	-

The basic genotype of strains used in prior transporter assay studies is provided.

Importantly, research has been unable to experimentally identify the highly efficient pentose transport system of *P. stipitis*, even though it is the source organism for many pentose metabolic genes. Specifically, a *P. stipitis *genomic DNA library transformed into a transporter-null strain of *S. cerevisiae *yielded no transformants able to grow on xylose [64]. Further investigation is needed because *P. stipitis *is known to have an excellent xylose transport system, yet to date no high-affinity xylose transporters have been isolated. The recently published genome sequence of this organism will certainly aid future research in this area [76], although most transporter proteins in the genome are currently annotated by homology and have not been characterized.

All of these studies make up a large body of work concerning heterologous transport protein expression in *S. cerevisiae*, and motivate further exploration for dedicated xylose transporters. However, future studies in this area must proceed with caution. Owing to the native transport characteristics of yeast, efficient transporters may need to be discovered through the use HXT family knockout strains [77]. It is also unknown how transporter proteins interact with each other to produce certain phenotypes. Specifically, *P. stipitis *xylose transport may involve transporter cooperation to achieve a high rate of xylose influx. Evidence of such transporter cooperation has already been demonstrated when coexpressing *C. intermedia GXS1 *and *GXF1 *[73]. Additionally, many transmembrane proteins act as sensors; for example, *RGT2 *and can control transporter expression [78]. Furthermore, the internal xylose catabolism pathway used in conjunction with heterologous or engineered xylose transporters can determine the level of flux control exhibited by the transport step [67]. Naturally, as the internal metabolic pathway is improved, pentose transport will become a greater limitation. Therefore, novel tools and approaches must accomplish simultaneous optimization of transport and metabolism - the tools and approaches of panmetabolic engineering.

### Panmetabolic engineering: the path forward for pentose utilization in yeast

As demonstrated above, pathway and transport optimization strategies have had little crossover. Pathway optimization targets were shown to have moved beyond merely expressing initial pathway genes to include those encoding proteins in the pentose phosphate pathway. These modifications greatly improved xylose assimilation and fermentation rates, yet, the most advanced strains used in the transporter expression studies (TMB3201 and H2219) only contained simple complementation of the three-enzyme xylose oxidoreductase pathway. We propose that this dichotomy exists because pathway-engineering approaches begin with the assumption that internal cellular pathways limit maximum production rates. As a result, approaches focus on importing heterologous pathways, and subsequently removing intracellular flux bottlenecks by replacing low capacity enzymes with overexpressed or optimized versions [28,79]. Despite this, strains with these improvements still exhibit suboptimal growth rates on xylose and arabinose [45,51]. Regardless, these strictly intracellular pathway engineering approaches will fail when the assumption is incorrect; that is, when molecular transport limits maximum production rates. This premise is the motivation for heterologous transporter expression. However, the vast majority of transporter studies have only investigated how to obtain improved transport through heterologous transporter expression; pathway fitness is generally not considered. From these points of view, the inter-related problems of pathway inadequacy and transport limitation have been studied in isolation. Therefore, to achieve the optimal biofuel-producing cell, novel pathway engineering and molecular transport engineering approaches must be combined and simultaneously optimized.

Therefore, the field seems poised for a new development: panmetabolic engineering. In this paradigm, the transport and pathway steps comprising the whole utilization pathway will be optimized concurrently, and may result in leaps forward in biofuel production from lignocellulosic biomass. In this regard, the expression and activity profiles across the entire pathway will be coordinated for maximal flux.

To implement panmetabolic engineering, new tools will be necessary. These tools must be able to simultaneously evaluate the effectiveness of individual mutant proteins while balancing the expression of transporters and multiple pathway genes. At the moment, nearly all protein and metabolic engineering strategies proceed in a stepwise manner to improve pathways. A recent example of complex, multistep pathway optimization is seen with improving levopimaradiene production in *E. coli *[80]. As a result, high-throughput methods involving combinatorial engineering of transporters and downstream enzymes are necessary to accelerate pathway improvement. One such approach could be achieved by adapting existing tools such as DNA assembler [10]. This tool takes advantage of yeast homologous recombination to build multiplex gene assembly and plasmid construction without restriction enzyme cloning, and can be used to randomize genes and promoters. Thus, mixtures of transporter and pathway enzyme mutants along with expression elements could be transformed into yeast to allow for simultaneous gene and expression level selection. Other approaches are certainly possible, and will largely be informed by the ever-increasing list of combinatorial pathway modification strategies [81]. Particular challenges with such approaches include search space reduction and improved selection strategies.

Bioinformatic tools can help pre-select (or potentially, may one day design *de novo*) enzymes of interest for the desired pathway. Protein structural information can help limit search spaces for directed evolution or rational design strategies. Resources such as the Basic Local Alignment Search Tool (BLAST) draw upon the success of genomic sequencing projects [76,82], allowing access to the extant biodiversity in other organisms. An example of the power of structural information and homology was shown in the recent engineering of levopimaradiene synthase [80]. By combining protein engineering with upstream pathway modifications, a 2,600-fold increase in levopimaradiene production was observed [80]. In terms of pentose utilization, enzyme sequence homology among organisms was used to assemble the *L. plantarum *arabinose pathway in yeast [51], to discover a functional heterologous bacterial XI [48], and to find likely candidates for pentose transport capability. Increasing sequence information from organisms relevant to lignocellulosic biomass utilization (including organism such as *P. stipitis *and *T. reesei) *can advance the panmetabolic engineering approach by providing more diversity of starting pathway proteins.

However, a high-throughput method will eventually be necessary to experimentally validate the functions of sequenced genes. Screening strategies (often employed to enable directed evolution) may be useful, but a high-throughput genotype to phenotype mapping method such as multiscale analysis of library enrichment (SCALEs) [83] may work best. With this approach, individual enzymes or whole operons that confer beneficial phenotypes may be discovered. With advancements in microarray technology, an adapted form of SCALEs could be used to screen a genomic library in yeast for transporter, pathway and regulatory enzymes. Such an approach could identify novel enzymes that confer beneficial phenotypes but do not yet have an annotated 'relative' and therefore are not identifiable using *in silico *search strategies such as BLAST. High-throughput phenotyping and analysis will not only be useful for bioprospecting efforts, but for screening the large libraries created from the large-scale combinatorial techniques mentioned above.

Classic strain engineering methods such as evolutionary engineering have been, and will probably continue to be, effective in bringing about efficient pentose metabolism in *S. cerevisiae. *Thus, the role of evolutionary engineering combined with panmetabolic engineering must be considered. Evolutionary engineering has been intensively used in industry for obtaining microorganisms with desired properties, such as efficient utilization of substrate and higher product tolerance [84]. Recently, evolutionary adaptation of engineered *S. cerevisiae *showed significant improvements in growth rate, utilization of pentose sugars and ethanol production [61,68,85-88]. However, this process should be directed and accelerated to more robustly harness the power of natural selection to evolve *S. cerevisiae *strains with the desired properties for ethanol production from lignocellulosic biomass. Additionally, advances in high-throughput genome sequencing and detection of single nucleotide polymorphisms have begun to enable the resequencing of evolved strains. Such capability would speed the development of rational techniques for strain improvement by enabling a link between genotype and phenotype. Already, combining the power of evolution with rational bioinformatics and systems biology has produced iterative strain engineering [89]. As these techniques develop, combining evolutionary engineering with pathway construction can further increase yields and efficiencies. However, computational paradigms will be necessary to draw the complete link between mutant genome sequence and functional phenotype.

Directed evolution of proteins has been used for redesigning and optimizing target enzymes and has obtained significant improvements in stability, tolerance, substrate specificity and product selectivity [90], although this technique has been underused in engineering transmembrane proteins [91]. For successful biofuel production with pentose sugars, directed evolution could be used at several points in the pentose assimilation process: for optimizing enzyme properties (such as higher activity of XI, low selectivity of XR to NADPH and optimal activity of XKS), for increasing transporter specificity towards xylose and arabinose, and for improving tolerance to ethanol. By combining directed evolution with more global evolutionary engineering strategies, it may be possible to simultaneously co-evolve multiple proteins in this process. Thus, this vision of panmetabolic engineering would prove powerful for improving metabolic flux in the context of lignocellulosic biomass utilization.

### Global cellular tools for improving non-pathway based phenotypes

Global approaches emerging from synthetic and systems biology may also have a role in improving pentose pathways [92]. Particularly, enhanced understanding of cellular signaling and synthetic circuitry could play a large role in optimization of pentose utilization. Lessons can be learned from other successful metabolic synthetic biology projects [93], yet the singular problem of exogenous sugar utilization will necessitate panmetabolic engineering along with global, synthetic cellular engineering.

Catabolite sensing may limit pentose metabolism, although mechanisms could be optimized to improve the fermentation characteristics of modified yeasts. Initial work has begun on understanding the regulatory effects of heterologous pathway expression [78], but further investigation must be undertaken. Glucose sensing and repression plays a large role in the metabolic response of yeast [94-98], including regulation of the hexokinase *HXK2 *in response to glucose [99]. Additionally, the yeast hexose transporters are subject to glucose regulation through a variety of mechanisms [100]. Furthermore, recent discoveries point to additional regulatory mechanisms triggered by glucose, such as cytosolic pH, influencing global cellular metabolic phenotypes [101]. Finally, transmembrane sensor proteins are thought to initiate several responses to extracellular metabolites. In many cases, these are major facilitator superfamily proteins with strong similarity to transport-capable transmembrane proteins. Future work must investigate the differences between a sensor and a transporter.

All of these regulatory mechanisms may play a role in the diauxic growth phenotype exhibited when yeast are presented with two carbon sources. Indeed, glucose-activated repression of the PPP may contribute to the low flux phenotypes observed in hexose and pentose cofermentations [78]. Optimization of this regulatory network must take place for panmetabolic engineering to succeed. For direction, reprogramming motility and toxin degradation in bacteria [102] demonstrates the feasibility of regulatory reprogramming, which may permit more efficient hexose and pentose consumption.

In addition to catabolite sensing and response, other global cellular phenotypes such as product tolerance have a large influence on the success of the fermentation. Importantly, improving cellular tolerances is possible, although underused [103]. In both of these applications, next generation metabolic engineering approaches such as global transcription machinery engineering (gTME) [103] may be able to alter global characteristics of *S. cerevisiae*. Without relying on *a priori *knowledge, gTME applies the success of directed evolution strategies to transcription factors. The major premise of this approach is that introducing dominant mutant alleles of generic transcription-related proteins can reprogram gene networks and cellular metabolism. In particular, key transcription factors (such as the TATA binding protein in yeast) are mutated to create diverse libraries. These mutants are selected on the basis of their ability to improve cellular phenotypes. By doing so, it is possible to alter complex phenotypes such as ethanol tolerance in yeast [103]. Using this method, transcription factors may also be engineered to optimize cellular systems for improved pentose fermentation characteristics, because the metabolism of sugars is tightly regulated [96,98]. This approach has the ability to improve several of the phenotypes related to pentose utilization, including high sugar tolerance and regulatory issues. Collectively, whole cell approaches may integrate the utilization pathway more fully into the host, and may alleviate difficulties such as cofactor imbalances and regulation.

## Conclusion

Metabolic engineering approaches must be adapted to address the challenges for pathway and global cellular optimization that currently limit the construction of an integrated, efficient pentose pathway. Although much progress has indeed been made in the past 20 years towards lignocellulosic biomass conversion by yeasts, the iterative approach of identifying limiting steps, optimizing and identifying limiting steps, *ad infinitum*, must accelerate. More emphasis on host genome and regulatory structure must occur in future projects to understand the full effect of biological complexity on a pathway. In this regard, classic approaches combined with next-generation technologies may be combined to allow simultaneous optimization of all steps in a pathway. The idealized approach combines the power of many approaches including pathway engineering, directed evolution, evolutionary engineering, and combinatorial genetics to harness this cellular complexity. To date, many of these approaches have been performed in isolation for creating pentose-utilizing yeast. In summary, the field stands ready for the paradigm shift to panmetabolic engineering, necessarily including novel global cellular engineering tools.

## Competing interests

The authors declare that they have no competing interests.

## Authors' contributions

EY, SL and HA conceptualized the manuscript. EY prepared the sections on xylose transport and SL prepared the sections on catabolic pathways. All authors contributed to the introduction, conclusions and panmetabolic engineering sections. HA provided crucial review and contributed to the writing and editing of all sections. All authors read and approved the final manuscript.

## References

[B1] Farrell AE Plevin RJ Turner BT Jones AD O'Hare M Kammen DM Ethanol can contribute to energy and environmental goals Science 2006 311 5760 506 508 10.1126/science.1121416 16439656

[B2] Alper H Stephanopoulos G Engineering for biofuels: exploiting innate microbial capacity or importing biosynthetic potential? Nature Reviews Microbiology 2009 7 10 715 723 10.1038/nrmicro2186 19756010

[B3] Bailey JE Toward a science of metabolic engineering Science 1991 252 5013 1668 1675 10.1126/science.2047876 2047876

[B4] Stephanopoulos G Vallino JJ Network rigidity and metabolic engineering in metabolite overproduction Science 1991 252 5013 1675 1681 10.1126/science.1904627 1904627

[B5] Wiechert W C-13 metabolic flux analysis Metabolic Engineering 2001 3 3 195 206 10.1006/mben.2001.0187 11461141

[B6] Shintani D DellaPenna D Elevating the vitamin E content of plants through metabolic engineering Science 1998 282 5396 2098 2100 10.1126/science.282.5396.2098 9851934

[B7] Madison LL Huisman GW Metabolic engineering of poly(3-hydroxyalkanoates): from DNA to plastic Microbiology and Molecular Biology Reviews 1999 63 1 21 53 10066830 10.1128/mmbr.63.1.21-53.1999 PMC98956

[B8] Varma A Palsson BO Metabolic flux balancing - basic concepts, scientific and practical use Bio-Technology 1994 12 10 994 998

[B9] Martin CH Nielsen DR Solomon KV Prather KLJ Synthetic metabolism: engineering biology at the protein and pathway scales Chemistry & Biology 2009 16 3 277 286 10.1016/j.chembiol.2009.01.010 19318209

[B10] Shao ZY Zhao H Zhao HM DNA assembler: an in vivo genetic method for rapid construction of biochemical pathways Nucleic Acids Research 2009 37 2 e16 10.1093/nar/gkn991 19074487 PMC2632897

[B11] Nair NU Zhao HM Mutagenic inverted repeat assisted genome engineering (MIRAGE) Nucleic Acids Research 2009 37 1 10.1093/nar/gkn943 19050015 PMC2615605

[B12] Beisel CL Smolke CD Design principles for riboswitch function PLoS Computational Biology 2009 5 4 e1000363 10.1371/journal.pcbi.1000363 19381267 PMC2666153

[B13] Haseltine EL Arnold FH Implications of rewiring bacterial quorum sensing Applide and Environmental Microbiology 2008 74 2 437 445 10.1128/AEM.01688-07 PMC2223271 18039819

[B14] Atsumi S Hanai T Liao JC Non-fermentative pathways for synthesis of branched-chain higher alcohols as biofuels Nature 2008 451 7174 86 89 10.1038/nature06450 18172501

[B15] Lee PC Schmidt-Dannert C Metabolic engineering towards biotechnological production of carotenoids in microorganisms Applied Microbiology and Biotechnology 2002 60 1-2 1 11 10.1007/s00253-002-1101-x 12382037

[B16] Schmidt-Dannert C Umeno D Arnold FH Molecular breeding of carotenoid biosynthetic pathways Nature Biotechnology 2000 18 7 750 753 10.1038/77319 10888843

[B17] Anthony JR Anthony LC Nowroozi F Kwon G Newman JD Keasling JD Optimization of the mevalonate-based isoprenoid biosynthetic pathway in *Escherichia coli *for production of the anti-malarial drug precursor amorpha-4,11-diene Metabolic Engineering 2009 11 1 13 19 10.1016/j.ymben.2008.07.007 18775787

[B18] Hawkins KM Smolke CD Production of benzylisoquinoline alkaloids in *Saccharomyces cerevisiae* Nature Chemical Biology 2008 4 9 564 573 10.1038/nchembio.105 18690217 PMC2830865

[B19] Atsumi S Cann AF Connor MR Shen CR Smith KM Brynildsen MP Chou KJY Hanai T Liao JC Metabolic engineering of *Escherichia coli *for 1-butanol production Metabolic Engineering 2008 10 6 305 311 10.1016/j.ymben.2007.08.003 17942358

[B20] Yu H Tyo K Alper H Klein-Marcuschamer D Stephanopoulos G A high-throughput screen for hyaluronic acid accumulation in recombinant *Escherichia coli *transformed by libraries of engineered sigma factors Biotechnology and Bioengineering 2008 101 4 788 796 10.1002/bit.21947 18500764

[B21] Alper H Stephanopoulos G Uncovering the gene knockout landscape for improved lycopene production in *E. coli* Applied Microbiology and Biotechnology 2008 78 5 801 810 10.1007/s00253-008-1373-x 18239914

[B22] Stephanopoulos G Challenges in engineering microbes for biofuels production Science 2007 315 5813 801 804 10.1126/science.1139612 17289987

[B23] Atsumi S Liao JC Metabolic engineering for advanced biofuels production from *Escherichia coli* Current Opinion in Biotechnology 2008 19 5 414 419 10.1016/j.copbio.2008.08.008 18761088 PMC2673505

[B24] Lee SK Chou H Ham TS Lee TS Keasling JD Metabolic engineering of microorganisms for biofuels production: from bugs to synthetic biology to fuels Current Opinion in Biotechnology 2008 19 6 556 563 10.1016/j.copbio.2008.10.014 18996194

[B25] Van Vleet JH Jeffries TW Yeast metabolic engineering for hemicellulosic ethanol production Current Opinion in Biotechnology 2009 20 3 300 306 10.1016/j.copbio.2009.06.001 19545992

[B26] Hahn-Hagerdal B Karhumaa K Fonseca C Spencer-Martins I Gorwa-Grauslund MF Towards industrial pentose-fermenting yeast strains Applied Microbiology and Biotechnology 2007 74 5 937 953 10.1007/s00253-006-0827-2 17294186

[B27] Hahn-Hagerdal B Galbe M Gorwa-Grauslund MF Liden G Zacchi G Bio-ethanol - the fuel of tomorrow from the residues of today Trends in Biotechnology 2006 24 12 549 556 10.1016/j.tibtech.2006.10.004 17050014

[B28] Ho N Chen Z Brainard A Genetically engineered *Saccharomyces *yeast capable of effective cofermentation of glucose and xylose Applied Environmental Microbiology 1998 64 1852 1859 10.1128/aem.64.5.1852-1859.1998 PMC106241 9572962

[B29] Richard P Toivari MH Penttilä M Evidence that the gene YLR070c of *Saccharomyces cerevisiae *encodes a xylitol dehydrogenase FEBS Letters 1999 457 1 135 138 10.1016/S0014-5793(99)01016-9 10486580

[B30] Traff K Jonsson L Hahn-Hagerdal B Putative xylose and arabinose reductases in *Saccharomyces cerevisiae* Yeast 2002 19 1233 1241 10.1002/yea.913 12271459

[B31] Richard P Toivari MH Penttilä M The role of xylulokinase in *Saccharomyces cerevisiae *xylulose catabolism FEMS Microbiology Letters 2000 190 1 39 43 10.1111/j.1574-6968.2000.tb09259.x 10981687

[B32] Wenger JW Schwartz K Sherlock G Bulk segregant analysis by high-throughput sequencing reveals a novel xylose utilization gene from *Saccharomyces cerevisiae* PLoS Genetics 6 5 e1000942 10.1371/journal.pgen.1000942 20485559 PMC2869308

[B33] Matsushika A Inoue H Kodaki T Sawayama S Ethanol production from xylose in engineered *Saccharomyces cerevisiae *strains: current state and perspectives Applied Microbiology and Biotechnology 2009 84 1 37 53 10.1007/s00253-009-2101-x 19572128

[B34] van Maris A Winkler A Kuyper M de Laat W van Dijken J Pronk J Development of efficient xylose fermentation in *Saccharomyces cerevisiae*: xylose isomerase as a key component Advances in Biochemical Engineering/Biotechnology 2007 179 204 full_text 17846724 10.1007/10_2007_057

[B35] Jeffries TW Engineering yeasts for xylose metabolism Current Opinion in Biotechnology 2006 17 3 320 326 10.1016/j.copbio.2006.05.008 16713243

[B36] Hahn-Hagerdal B Karhumaa K Jeppsson M Gorwa-Grauslund M Metabolic engineering for pentose utilization in *Saccharomyces cerevisiae* Advances in Biochemical Engineering/Biotechnology 2007 108 147 177 full_text 17846723 10.1007/10_2007_062

[B37] Richard P Verho R Putkonen M Londesborough J Penttila M Production of ethanol from L-arabinose by *Saccharomyces cerevisiae *containing a fungal L-arabinose pathway FEMS Yeast Research 2003 3 2 185 189 10.1016/S1567-1356(02)00184-8 12702451

[B38] Kotter P Amore R Hollenberg CP Ciriacy M Isolation and characterization of the *Pichia stipitis *xylitol dehydrogenase gene, *XYL2*, and construction of a xylose-utilizing *Saccharomyces cerevisiae *transformant Current Genetics 1990 18 6 493 500 10.1007/BF00327019 2127555

[B39] Walfridsson M Bao XM Anderlund M Lilius G Bulow L Hahn-Hagerdal B Ethanolic fermentation of xylose with *Saccharomyces cerevisiae *harboring the *Thermus thermophilus xylA *gene, which expresses an active xylose (glucose) isomerase Applied and Environmental Microbiology 1996 62 12 4648 4651 8953736 10.1128/aem.62.12.4648-4651.1996 PMC168291

[B40] Becker J Boles E A modified *Saccharomyces cerevisiae *strain that consumes L-arabinose and produces ethanol Applied and Environmental Microbiology 2003 69 7 4144 4150 10.1128/AEM.69.7.4144-4150.2003 12839792 PMC165137

[B41] Nevoigt E Progress in metabolic engineering of *Saccharomyces cerevisiae* Microbiology and Molecular Biology Reviews 2008 72 3 379 412 10.1128/MMBR.00025-07 18772282 PMC2546860

[B42] Richard P Putkonen M Vaananen R Londesborough J Penttila M The missing link in the fungal L-arabinose catabolic pathway, identification of the L-xylulose reductase gene Biochemistry 2002 41 20 6432 6437 10.1021/bi025529i 12009906

[B43] Richard P Londesborough J Putkonen M Kalkkinen N Penttila M Cloning and expression of a fungal L-arabinitol 4-dehydrogenase gene Journal of Biological Chemistry 2001 276 44 40631 40637 10.1074/jbc.M104022200 11514550

[B44] van Maris A Abbott D Bellissimi E van den Brink J Kuyper M Luttik M Wisselink H Scheffers W van Dijken J Pronk J Alcoholic fermentation of carbon sources in biomass hydrolysates by *Saccharomyces cerevisiae*: current status Antonie van Leeuwenhoek 2006 90 4 391 418 10.1007/s10482-006-9085-7 17033882

[B45] Jin YS Alper H Yang YT Stephanopoulos G Improvement of xylose uptake and ethanol production in recombinant *Saccharomyces cerevisiae *through an inverse metabolic engineering approach Applied and Environmental Microbiology 2005 71 12 8249 8256 10.1128/AEM.71.12.8249-8256.2005 16332810 PMC1317456

[B46] Bettiga M Hahn-Hagerdal B Gorwa-Grauslund M Comparing the xylose reductase/xylitol dehydrogenase and xylose isomerase pathways in arabinose and xylose fermenting *Saccharomyces cerevisiae *strains Biotechnology for Biofuels 2008 1 1 16 10.1186/1754-6834-1-16 18947407 PMC2579915

[B47] Gardonyi M Hahn-Hagerdal B The *Streptomyces rubiginosus *xylose isomerase is misfolded when expressed in *Saccharomyces cerevisiae* Enzyme and Microbial Technology 2003 32 2 252 259 10.1016/S0141-0229(02)00285-5

[B48] Brat D Boles E Wiedemann B Functional expression of a bacterial xylose isomerase in *Saccharomyces cerevisiae* Applied and Environmental Microbiology 2009 75 8 2304 2311 10.1128/AEM.02522-08 19218403 PMC2675233

[B49] Kuyper M Harhangi HR Stave AK Winkler AA Jetten MSM de Laat W den Ridder JJJ Op den Camp HJM van Dijken JP Pronk JT High-level functional expression of a fungal xylose isomerase: the key to efficient ethanolic fermentation of xylose by *Saccharomyces cerevisiae*? FEMS Yeast Research 2003 4 1 69 78 10.1016/S1567-1356(03)00141-7 14554198

[B50] Madhavan A Tamalampudi S Ushida K Kanai D Katahira S Srivastava A Fukuda H Bisaria VS Kondo A Xylose isomerase from polycentric fungus *Orpinomyces*: gene sequencing, cloning, and expression in *Saccharomyces cerevisiae *for bioconversion of xylose to ethanol Applied Microbiology and Biotechnology 2009 82 6 1067 1078 10.1007/s00253-008-1794-6 19050860

[B51] Wisselink H Toirkens M del Rosario Franco Berriel M Winkler A van Dijken J Pronk J van Maris A Engineering of *Saccharomyces cerevisiae *for efficient anaerobic alcoholic fermentation of L-arabinose Applied and Environmental Microbiology 2007 73 4881 4891 10.1128/AEM.00177-07 17545317 PMC1951023

[B52] Santos dos MM Raghevendran V Kötter P Olsson L Nielsen J Manipulation of malic enzyme in *Saccharomyces cerevisiae *for increasing NADPH production capacity aerobically in different cellular compartments Metabolic Engineering 2004 6 4 352 363 10.1016/j.ymben.2004.06.002 15491864

[B53] Jeffries TW Jin YS Metabolic engineering for improved fermentation of pentoses by yeasts Applied Microbiology and Biotechnology 2004 63 5 495 509 10.1007/s00253-003-1450-0 14595523

[B54] Wiedemann B Boles E Codon-optimized bacterial genes improve L-arabinose fermentation in recombinant *Saccharomyces cerevisiae* Applied and Environmental Microbiology 2008 74 7 2043 2050 10.1128/AEM.02395-07 18263741 PMC2292587

[B55] Jeppsson M Bengtsson O Franke K Lee H Hahn-Hagerdal B Gorwa-Grauslund M The expression of a *Pichia stipitis *xylose reductase mutant with higher K_m _for NADPH increases ethanol production from xylose in recombinant *Saccharomyces cerevisiae* Biotechnology and Bioengineering 2006 93 665 673 10.1002/bit.20737 16372361

[B56] Watanabe S Abu Saleh A Pack SP Annaluru N Kodaki T Makino K Ethanol production from xylose by recombinant *Saccharomyces cerevisiae *expressing protein-engineered NADH-preferring xylose reductase from *Pichia stipitis* Microbiology 2007 153 9 3044 3054 10.1099/mic.0.2007/007856-0 17768247

[B57] Karhumaa K Fromanger R Hahn-Hagerdal B Gorwa-Grauslund M High activity of xylose reductase and xylitol dehydrogenase improves xylose fermentation by recombinant *Saccharomyces cerevisiae* Applied Microbiology and Biotechnology 2007 73 1039 1046 10.1007/s00253-006-0575-3 16977466

[B58] Bellissimi E Dijken JP Pronk JT Maris AJA Effects of acetic acid on the kinetics of xylose fermentation by an engineered, xylose-isomerase-based *Saccharomyces cerevisiae *strain FEMS Yeast Research 2009 9 3 358 364 10.1111/j.1567-1364.2009.00487.x 19416101

[B59] Chiang LC Hsiao HY Ueng PP Tsao GT Enzymatic and microbial preparation of D-xylulose from D-xylose Applied and Environmental Microbiology 1981 42 1 66 69 16345816 10.1128/aem.42.1.66-69.1981 PMC243963

[B60] Jeffries T Utilization of xylose by bacteria, yeasts, and fungi Advances in Biochemical Engineering/Biotechnology 1983 27 1 32 full_text 6437152 10.1007/BFb0009101

[B61] Madhavan A Tamalampudi S Srivastava A Fukuda H Bisaria V Kondo A Alcoholic fermentation of xylose and mixed sugars using recombinant *Saccharomyces cerevisiae *engineered for xylose utilization Applied Microbiology and Biotechnology 2009 82 6 1037 1047 10.1007/s00253-008-1818-2 19125247

[B62] Heath EC Horecker BL Smyrniotis PZ Takagi Y Pentose fermentation by *Lactobacillus planarum* Journal of Biological Chemistry 1958 231 2 1031 1037 13539034

[B63] Wang Q Zhang Y Yang C Xiong H Lin Y Yao J Li H Xie L Zhao W Yao Y Acetylation of metabolic enzymes coordinates carbon source utilization and metabolic flux Science 2010 327 5968 1004 1007 10.1126/science.1179687 20167787 PMC4183141

[B64] Hamacher T Becker J Gardonyi M Hahn-Hagerdal B Boles E Characterization of the xylose-transporting properties of yeast hexose transporters and their influence on xylose utilization Microbiology 2002 148 Pt 9 2783 2788 12213924 10.1099/00221287-148-9-2783

[B65] Sedlak M Ho NWY Characterization of the effectiveness of hexose transporters for transporting xylose during glucose and xylose co-fermentation by a recombinant *Saccharomyces *yeast Yeast 2004 21 8 671 684 10.1002/yea.1060 15197732

[B66] Saloheimo A Rauta J Stasyk OV Sibirny AA Penttila M Ruohonen L Xylose transport studies with xylose-utilizing *Saccharomyces cerevisiae s*trains expressing heterologous and homologous permeases Applied Microbiology and Biotechnology 2007 74 5 1041 1052 10.1007/s00253-006-0747-1 17180689

[B67] Gardonyi M Jeppsson M Liden G Gorwa-Grausland MF Hahn-Hagerdal B Control of xylose consumption by xylose transport in recombinant *Saccharomyces cerevisiae* Biotechnology and Bioengineering 2003 82 7 818 824 10.1002/bit.10631 12701148

[B68] Kuyper M Toirkens MJ Diderich JA Winkler AA van Dijken JP Pronk JT Evolutionary engineering of mixed-sugar utilization by a xylose-fermenting *Saccharomyces cerevisiae *strain FEMS Yeast Research 2005 5 10 925 934 10.1016/j.femsyr.2005.04.004 15949975

[B69] Wahlbom CF Otero RRC van Zyl WH Hahn-Hagerdal B Jonsson LJ Molecular analysis of a *Saccharomyces cerevisiae *mutant with improved ability to utilize xylose shows enhanced expression of proteins involved in transport, initial xylose metabolism, and the pentose phosphate pathway Applied and Environmental Microbiology 2003 69 2 740 746 10.1128/AEM.69.2.740-746.2003 12570990 PMC143595

[B70] Pao SS Paulsen IT Saier MH Major facilitator superfamily Microbiology and Molecular Biology Reviews 1998 62 1 1 34 9529885 10.1128/mmbr.62.1.1-34.1998 PMC98904

[B71] Leandro MJ Fonseca C Goncalves P Hexose and pentose transport in ascomycetous yeasts: an overview FEMS Yeast Research 2009 9 4 511 525 10.1111/j.1567-1364.2009.00509.x 19459982

[B72] Leandro MJ Goncalves P Spencer-Martins I Two glucose/xylose transporter genes from the yeast *Candida intermedia*: first molecular characterization of a yeast xylose-H^+ ^symporter Biochemical Journal 2006 395 543 549 10.1042/BJ20051465 16402921 PMC1462686

[B73] Leandro MJ Spencer-Martins I Goncalves P The expression in *Saccharomyces cerevisiae *of a glucose/xylose symporter from *Candida intermedia *is affected by the presence of a glucose/xylose facilitator Microbiology 2008 154 Pt 6 1646 1655 10.1099/mic.0.2007/015511-0 18524919

[B74] Runquist D Fonseca C Radstrom P Spencer-Martins I Hahn-Hagerdal B Expression of the Gxf1 transporter from *Candida intermedia *improves fermentation performance in recombinant xylose-utilizing *Saccharomyces cerevisiae* Applied Microbiology and Biotechnology 2009 82 1 123 130 10.1007/s00253-008-1773-y 19002682

[B75] Hector RE Qureshi N Hughes SR Cotta MA Expression of a heterologous xylose transporter in a *Saccharomyces cerevisiae *strain engineered to utilize xylose improves aerobic xylose consumption Applied Microbiology and Biotechnology 2008 80 4 675 684 10.1007/s00253-008-1583-2 18629494

[B76] Jeffries TW Grigoriev IV Grimwood J Laplaza JM Aerts A Salamov A Schmutz J Lindquist E Dehal P Shapiro H Genome sequence of the lignocellulose-bioconverting and xylose-fermenting yeast *Pichia stipitis* Nature Biotechnology 2007 25 319 326 10.1038/nbt1290 17334359

[B77] Wieczorke R Krampe S Weierstall T Freidel K Hollenberg CP Boles E Concurrent knock-out of at least 20 transporter genes is required to block uptake of hexoses in *Saccharomyces cerevisiae* FEBS Letters 1999 464 3 123 128 10.1016/S0014-5793(99)01698-1 10618490

[B78] Salusjarvi L Kankainen M Soliymani R Pitkanen JP Penttila M Ruohonen L Regulation of xylose metabolism in recombinant *Saccharomyces cerevisiae* Microbial Cell Factories 2008 7 16 10.1186/1475-2859-7-18 18533012 PMC2435516

[B79] Kotter P Ciriacy M Xylose fermentation by *Saccharomyces cerevisiae* Applied Microbiology and Biotechnology 1993 38 6 776 783 10.1007/BF00167144

[B80] Leonard E Ajikumar PK Thayer K Xiao WH Mo JD Tidor B Stephanopoulos G Prather KLJ Combining metabolic and protein engineering of a terpenoid biosynthetic pathway for overproduction and selectivity control Proceedings of the National Academy of Sciences of the United States of America 2010 107 31 13654 13659 10.1073/pnas.1006138107 20643967 PMC2922259

[B81] Santos CNS Stephanopoulos G Combinatorial engineering of microbes for optimizing cellular phenotype Current Opinion in Chemical Biology 2008 12 2 168 176 10.1016/j.cbpa.2008.01.017 18275860

[B82] Martinez D Berka RM Henrissat B Saloheimo M Arvas M Baker SE Chapman J Chertkov O Coutinho PM Cullen D Genome sequencing and analysis of the biomass-degrading fungus *Trichoderma reesei *(syn. *Hypocrea jecorina*) Nature Biotechnology 2008 26 5 553 560 10.1038/nbt1403 18454138

[B83] Lynch MD Warnecke T Gill RT SCALEs: multiscale analysis of library enrichment Nature Methods 2007 4 1 87 93 10.1038/nmeth946 17099705

[B84] Wisselink HW Toirkens MJ Wu Q Pronk JT van Maris AJA Novel evolutionary engineering approach for accelerated utilization of glucose, xylose, and arabinose mixtures by engineered *Saccharomyces cerevisiae *strains Applied and Environmental Microbiology 2009 75 4 907 914 10.1128/AEM.02268-08 19074603 PMC2643596

[B85] Wahlbom C van Zyl W Jonsson L Hahn-Hagerdal B Otero R Generation of the improved recombinant xylose-utilizing *Saccharomyces cerevisiae *TMB 3400 by random mutagenesis and physiological comparison with *Pichia stipitis *CBS 6054 FEMS Yeast Res 2003 3 319 326 10.1016/S1567-1356(02)00206-4 12689639

[B86] Kuyper M Hartog MMP Toirkens MJ Almering MJH Winkler AA van Dijken JP Pronk JT Metabolic engineering of a xylose-isomerase-expressing *Saccharomyces cerevisiae *strain for rapid anaerobic xylose fermentation FEMS Yeast Research 2005 5 4-5 399 409 10.1016/j.femsyr.2004.09.010 15691745

[B87] Matsushika A Oguri E Sawayama S Evolutionary adaptation of recombinant shochu yeast for improved xylose utilization Journal of Bioscience and Bioengineering 2010 110 1 102 105 10.1016/j.jbiosc.2010.01.002 20541125

[B88] Garcia Sanchez R Karhumaa K Fonseca C Sanchez Nogue V Almeida J Larsson C Bengtsson O Bettiga M Hahn-Hagerdal B Gorwa-Grauslund M Improved xylose and arabinose utilization by an industrial recombinant *Saccharomyces cerevisiae *strain using evolutionary engineering Biotechnology for Biofuels 2010 3 1 13 10.1186/1754-6834-3-13 20550651 PMC2908073

[B89] Tyo KEJ Kocharin K Nielsen J Toward design-based engineering of industrial microbes Current Opinion in Microbiology 2010 13 3 255 262 10.1016/j.mib.2010.02.001 20226723 PMC2885540

[B90] Jäckel C Kast P Hilvert D Protein design by directed evolution Annual Review of Biophysics 2008 37 1 153 173 10.1146/annurev.biophys.37.032807.125832 18573077

[B91] Bayley H Jayasinghe L Functional engineered channels and pores Molecular Membrane Biology 2004 21 4 209 220 10.1080/09687680410001716853 15371010

[B92] Young E Alper H Synthetic biology: tools to design, build, and optimize cellular processes Journal of Biomedicine and Biotechnology 2010 130781 20150964 10.1155/2010/130781 PMC2817555

[B93] Cantone I Marucci L Iorio F Ricci MA Belcastro V Bansal M Santini S di Bernardo M di Bernardo D Cosma MP A yeast synthetic network for *in vivo *assessment of reverse-engineering and modeling approaches Cell 2009 137 1 172 181 10.1016/j.cell.2009.01.055 19327819

[B94] Johnston M Feasting, fasting and fermenting - glucose sensing in yeast and other cells Trends in Genetics 1999 15 1 29 33 10.1016/S0168-9525(98)01637-0 10087931

[B95] Rolland F Winderickx J Thevelein JM Glucose-sensing and -signalling mechanisms in yeast FEMS Yeast Research 2002 2 2 183 201 12702307 10.1111/j.1567-1364.2002.tb00084.x

[B96] Santangelo GM Glucose signaling in *Saccharomyces cerevisiae* Microbiology and Molecular Biology Reviews 2006 70 1 253 282 10.1128/MMBR.70.1.253-282.2006 16524925 PMC1393250

[B97] Westergaard SL Oliveira AP Bro C Olsson L Nielsen J A systems biology approach to study glucose repression in the yeast *Saccharomyces cerevisiae* Biotechnology and Bioengineering 2007 96 1 134 145 10.1002/bit.21135 16878332

[B98] Gancedo JM The early steps of glucose signalling in yeast FEMS Microbiology Reviews 2008 32 4 673 704 10.1111/j.1574-6976.2008.00117.x 18559076

[B99] Palomino A Herrero P Moreno F Tpk3 and Snf1 protein kinases regulate Rgt1 association with *Saccharomyces cerevisiae **HXK2 *promoter Nucleic Acids Research 2006 34 5 1427 1438 10.1093/nar/gkl028 16528100 PMC1401511

[B100] Ozcan S Johnston M Function and regulation of yeast hexose transporters Microbiology and Molecular Biology Reviews 1999 63 3 554 569 10477308 10.1128/mmbr.63.3.554-569.1999 PMC103746

[B101] Dechant R Binda M Lee SS Pelet S Winderickx J Peter M Cytosolic pH is a second messenger for glucose and regulates the PKA pathway through V-ATPase The EMBO Journa 2010 29 15 2515 2526 10.1038/emboj.2010.138 PMC2928683 20581803

[B102] Sinha J Reyes SJ Gallivan JP Reprogramming bacteria to seek and destroy an herbicide Nature Chemical Biology 6 6 464 470 10.1038/nchembio.369 20453864 PMC2873063

[B103] Alper H Moxley J Nevoigt E Fink GR Stephanopoulos G Engineering yeast transcription machinery for improved ethanol tolerance and production Science 2006 314 5805 1565 1568 10.1126/science.1131969 17158319

[B104] Minet M Dufour ME Lacroute F Complementation of *Saccharomyces cerevisiae *auxotrophic mutants by *Arabidopsis thaliana *cDNAs The Plant Journal 1992 2 3 417 422 1303803 10.1111/j.1365-313x.1992.00417.x

[B105] Weierstall T Hollenberg CP Boles E Cloning and characterization of three genes (*SUT1-3*) encoding glucose transporters of the yeast *Pichia stipitis* Molecular Microbiology 1999 31 3 871 883 10.1046/j.1365-2958.1999.01224.x 10048030

